# Association of socioeconomic status with overall overweight and central obesity in men and women: the French Nutrition and Health Survey 2006

**DOI:** 10.1186/1471-2458-9-215

**Published:** 2009-07-02

**Authors:** Michel Vernay, Aurelie Malon, Amivi Oleko, Benoit Salanave, Candice Roudier, Emmanuelle Szego, Valerie Deschamps, Serge Hercberg, Katia Castetbon

**Affiliations:** 1 Nutritional Surveillance and Epidemiology Unit (USEN), Institut de Veille Sanitaire, Université Paris 13, Conservatoire des Arts et Métiers, Bobigny, France; 2 Unité de Recherche en Epidémiologie Nutritionnelle, U557 Inserm, U1125 Inra, Cnam, UP13, CRNH Idf, Bobigny, France

## Abstract

**Background:**

Identification of subpopulations at high risk of overweight and obesity is crucial for prevention and management of obesity in different socioeconomic status (SES) categories. The objective of the study was to describe disparities in the prevalence of overweight and obesity across socioeconomic status (SES) groups in 18–74 year-old French adults.

**Methods:**

Analyses were based on a multistage stratified random sample of non-institutionalized adults aged 18–74-years-old from the French Nutrition and Health Survey (ENNS), a cross-sectional national survey carried out in 2006/2007. Collected data included measured anthropometry (weight, height and waist circumference (WC)), demographic and SES data (occupation, education and frequency of holiday trips as a marker of family income). SES factors associated with overweight (BMI ≥ 25) and central obesity (WC above gender-specific references) were identified using multiple logistic regression.

**Results:**

Almost half (49.3%) of French adults were overweight or obese and 16.9% were obese. In men, the risk of overall overweight or obesity was associated with occupation (p < 0.05), whereas the risk of central obesity was independently associated with occupation (p < 0.05) and frequency of holiday trips (p < 0.01). In women, both overall and central overweight and obesity were independently associated with educational level (respectively p < 10^-3 ^and p < 10^-3^) and frequency of holiday trips (respectively p < 0.05 and p < 10^-3^).

**Conclusion:**

The prevalence of overweight and obesity was found to be similar to that of several neighbouring western European countries, and lower than the UK and eastern Europe. Risk of being overweight or obese varied across SES groups both in men and women, but associations were different between men and women, indicating differing determinants.

## Background

In France, as in neighbouring western Europe countries, the prevalence of obesity among adults is considered to be lower than that reported in the USA, Canada, the UK and eastern Europe [1,2]. However, since the end of the 1980s, France, like many developed countries, has had to face a rapid increase in the prevalence of overweight and obesity [3]. Several studies have highlighted the inverse association between socioeconomic status (SES) and risk of overweight and obesity in North American and northern European countries [4,5], where obesity and overweight are highly prevalent. Few data are available concerning overweight and obesity variations across SES in less affected countries such as France [6]. SES is commonly evaluated through occupational status, education level and income [7,8], corresponding to different potential individual mechanisms influencing lifestyle factors, such as leisure-time physical activity and food consumption.

In 2001, the French Ministry of Health implemented the French National Nutrition and Health Program (PNNS), whose main objective was to improve the health status of the population by influencing nutrition [9]. The PNNS has defined specific nutritional objectives for special groups, including the socially and financially disadvantaged. A national nutritional surveillance system was implemented at the onset of the PNNS. The monitoring system for overweight and obesity in the French general population was previously based mainly on self-reported data for adults and led to an underestimation of prevalence of overweight and obesity [3]. It is of particular interest to identify subpopulations at high risk of overweight or obesity in order to implement specially adapted programs for reducing obesity in different SES categories.

The objective of the present study was to describe socio-economic factors associated with overall and central overweight and obesity in French 18–74-year-old adults living in France in 2006–2007. Data were from the Nutrition and Health Survey (ENNS) that includes diet collection and nutritional status measurements [10].

## Methods

ENNS was a cross-sectional descriptive survey conducted from February 2006 to July 2007, based on a multistage stratified random sample of a non-institutionalized population living in France (excluding the island of Corsica and French overseas territories), aged 3–74-years-old. The study design included a food consumption survey and a health examination in children and adults.

### Sample design

Sample selection was based on a three-stage cluster design, stratified by eight large regions and by the degree of urbanization (four groups, from "rural" to "towns of more than 100,000 inhabitants" according to the classification of the French National Institute for Statistics and Economic Studies, INSEE). At the first sampling stage, 190 primary sampling units (i.e. boroughs or groups of boroughs) were selected. At the second sampling stage, households were drawn from phone listings. Phone numbers were randomly generated; the corresponding household location was verified in the 190 selected geographic zones, and professional and/or fax numbers were excluded. At the final stage, one subject per sampled household was selected using the birthday method, after verification of eligibility criteria. Criteria for adult eligibility were: subjects had to reside in mainland France during the period of the study; they had to be aged 18–74-years-old and spend at least five days a week in a "normal" household (as opposed to an institution, for example) with phone contact (landline or mobile phone); they were capable of understanding the reasons for the study; they were not artificially fed; and they agreed to participate in the study.

The survey protocol received the approval of the Ethical Committee (Hôpital Cochin n°2264), the Consultative Committee on Information Treatment of the Ministry of Research and the French Data Protection Authority (Commission Nationale de l'Informatique et des Libertés, CNIL, authorization n°905481).

### Anthropometric measurements

Body weight, height and waist circumference (WC) were measured at home or in a health examination centre of the French National Health Insurance System (CnamTS) by specifically trained physicians, nurses or dieticians using standardized protocols according to WHO recommendations [11]. Electronic digital scales (Seca^® ^Bellissima 841) were used to measure body weight with an accuracy of ± 100 g; the subject was lightly dressed (mainly underwear) and without shoes. Body height was measured to the nearest 0.5 cm using a portable stadiometer (Seca^® ^bodymeter 206). WC was measured using a flexible plastic fibre tape measure placed at the midpoint between the lower rib margin and the iliac crest.

### Socioeconomic and behavioural data

Socioeconomic and demographic data were obtained at home through face-to-face interviews using standardized questionnaires. Interviews included questions on age, marital status, area of residence, occupational status and education level. Participants were also asked if they had taken one or more holiday trips lasting four nights or more during the past twelve months, shown in previous studies as being strongly associated with household income [12].

Alcohol consumption was assessed using both the last seven-day-alcohol-frequency questionnaire, administered during the face-to-face interview, and information on alcohol consumption from three 24-h dietary recalls of the food consumption survey. Face-to-face interviews also included questions on smoking habits.

### Statistical analyses

Body mass index (BMI) was calculated as the ratio between weight (in kg) and height square (in meter). According to WHO references [11], subjects were classified as overweight/obese (BMI ≥ 25.0) or obese (BMI ≥ 30.0). According to EGIR recommendations [13], WC ≥ 94 cm in men and ≥ 80 cm in women was taken as a marker of central obesity. Prevalences were estimated in the sample with complete anthropometry data excluding pregnant women.

Occupational status was grouped into five categories: management and intermediate profession, self-employed and farmers, manual workers and employees, retired, homemakers and disabled persons; education was divided into four categories: university, high school, secondary school, primary school.

Alcohol consumption was divided into three categories: abstinence, moderate consumption (i.e. ≤ 30 g/d of ethanol for men, ≤ 20 g/d of ethanol for women) or heavy consumption (i.e. > 30 g/d for men, > 20 g/d for women).

Statistical analyses were carried out using Stata^® ^software (version 10; Stata Press, College Station, TX). The complex survey design and unequal probabilities of sample selection were taken into account using the "Svyset" procedure. For each gender, calibration was calculated according to national census data on age, educational diploma and whether or not the household included at least one child. The final weighting also included the period of measurement. Census data available on the web  were used to compare the characteristics of the weighted sample and the French general population. Univariate logistic regression was used to investigate the association of each SES marker and potential confounding variables with overall and central overweight and obesity. Factors associated with inclusion at alpha < 20% were included in the initial multivariate logistic model. Manual descending multivariate logistic regressions were carried out. Factors associated with inclusion at alpha < 5% remained in the final multivariate logistic model. Factors associated with inclusion at alpha ≥ 5% were retained in the final model when their exclusion led to an OR variation > 10%. Dummy categorical variables were used in logistic regression. Odds ratios (OR), 95% confidence intervals (CI_95_) and p-values (p) were calculated. Interactions between gender and several factors (birthplace, occupation, education, holidays and smoking habits for overweight and obesity, and area of residence and smoking habits for central obesity) suggested potential differing associations according to gender. Therefore, univariate and multivariate regression analyses were carried out separately for men and women.

## Results

### Characteristics of the subjects

Among 5,217 contacted eligible households, 3,115 adults aged 18–74 (1,126 men, 1,989 women) participated in the food consumption survey (59.7%) and 2,413 of them (876 men, 1,537 women) were measured for body height and weight (46.3%). In addition, 25 pregnant women were excluded; therefore 2,388 subjects were included in the present analyses. Compared to other degrees of urbanization, the participation rate was weaker in the Paris area (12.6% of households were from the Paris area, even though Paris area inhabitants represent 17% of the French households). Lack of time available for participating in data collection, along with only minor interest in nutrition, were the main reasons cited for refusal.

According to raw data, young adults and subjects with a low level of education were poorly represented in the sample of 2,388 subjects with complete anthropometry measurements (Table 1). Other sociodemographic characteristics were scarcely modified by the weighting procedure. Demographic and socioeconomic characteristics were mostly comparable between the weighted sample and the French general population, except for occupation. Demographic and socioeconomic characteristics were also comparable between men and women, except for occupation, alcohol consumption and smoking habits (Table 1).

**Table 1:** Social and demographic characteristics of the 2,388 adults aged 18–74 years (pregnant women excluded) with complete anthropometry measurements, compared to national census data, the French Nutrition and Health Survey (ENNS 2006–2007).

	Raw data		Weighted* data		Census data
			
	Men	Women	Men	Women	
Age (%) in years					
18–29	12.8	10.0	22.6	20.7	22.0
30–54	51.0	55.5	49.9	49.6	49.7
55–74	36.2	34.5	27.5	29.6	28.3
Birthplace (%)					
France (including overseas territories)	89.7	88.2	91.7	91.0	87.9
Europe	2.3	3.0	3.5	3.0	
Africa	6.2	4.9	4.4	4.6	12.1^§^
Other	0.7	1.2	0.4	1.4	
Missing	1.1	2.7	-	-	
Marital status (%)					
Married	63.0	57.1	60.6	57.7	54.8
Living together unmarried	10.7	13.8	11.4	14.8	34.1^₣^
Single	17.1	11.3	22.9	15.5	
Separated/divorced/widowed	9.2	17.8	5.1	11.9	11.1
At least one child at home (%)					
Yes	35.6	41.7	35.9	35.5	35.7
No	64.4	58.3	64.1	64.5	64.3
Occupation (%)					
Management/intermediate profession	30.1	33.7	20.9	16.6	20.2
Self-employed/farmers	6.1	2.6	5.3	2.4	5.2
Manual workers/employees	30.9	25.2	41.5	39.4	33.1
Retired	25.9	22.0	21.0	19.3	24.1
Homemakers, disabled persons, others	7.0	16.5	11.3	22.3	17.4
Education level (%)					
University	36.4	33.1	19.6	19.9	19.8
High school	18.9	19.2	17.3	17.2	17.2
Secondary school	35.0	34.1	44.7	40.3	42.5
Primary school	9.5	13.4	18.4	22.6	20.5
Missing	0.2	0.2	-	-	-
Holiday trip during the past 12 months (%)					
Yes	71.5	69.6	67.5	67.5	64.6^†^
No	27.0	37.8	32.5	32.5	35.4^†^
Missing	1.5	2.6	-	-	-
Area of residence (%)					
Rural	24.5	23.9	25.9	24.7	22.1^£^
[2,000–20,000]	18.7	15.8	18.4	16.1	19.3^£^
[20,000–100,000]	13.5	15.2	13.9	15.5	13.5^£^
[100,000–2,000,000]	31.6	32.0	32.7	32.4	28.6^£^
Paris area	11.7	13.1	9.1	11.4	16.5^£^
Alcohol consumption (%)					
Moderate	67.1	65.3	67.2	65.8	-
Abstainer	12.2	24.5	13.3	28.9	-
High	19.0	6.2	19.5	5.3	-
Missing	1.7	4.0	-	-	-
Smoking habits (%)					
Never-smoker	34.7	54.0	34.7	57.3	-
Current smoker	28.7	23.7	31.2	24.5	-
Former smoker	36.6	22.1	34.1	18.2	-
Missing	-	0.2	-	-	-

*. Weighting accounted for multilevel sampling design and for social and demographic characteristics compared to the national census (age, school diploma, household including at least one child or not).

^§^. Born outside France.

^₣^. Living together unmarried and single.

^†^. 15–79 y.

^£^. All population, children included.

Overall, 49.3% [46.4–52.1] of adults aged 18–74 were overweight, including obesity (BMI ≥ 25.0), and 16.9% [14.8–18.9] were strictly obese (BMI ≥ 30.0). The prevalence of overweight/obesity was higher in men (57.2% [52.6–61.7]) than in women (41.4% [38.1–44.7]), whereas the prevalence of obesity alone was similar in men (16.1% [12.9–19.3]) and women (17.6% [15.0–20.2]). Based on waist circumference, 46.9% [44.0–49.8] of adults aged 18–74 were considered to be centrally obese (≥ 94 cm in men, ≥ 80 cm in women). Women were more centrally obese (51.6% [48.1–55.1]) than men (42.3% [37.8–46.8]). Body height and weight were measured at a health examination centre for 50.6% of participants, and at home for 49.4%; mean anthropometric measurements were not statistically different according to the place where measurements were taken.

### Overall overweight and SES

Among the 2,388 subjects included in the health examination survey, 2,204 (831 men, 1,373 women) had complete data regarding anthropometry and SES characteristics (92.3%). According to raw data, sociodemographic and anthropometry characteristics were comparable between the 2,204 subjects with complete data and the 2,388 subjects who underwent anthropometric measurements (data not tabulated). In men, after adjustment for confounding variables [see Additional file 1], overall overweight was associated with occupation (p < 10^-3^). Compared to men classified into the management/intermediate professional category, self-employed and farmers had a significantly higher risk of being overweight independently of age. In contrast, the educational level was no longer significantly associated with overall overweight in multivariate analysis, whereas this was the case in univariate analysis. In multivariate analysis, overall obesity was not associated with SES characteristics (data not shown).

In women, education level (p < 10^-3^) and frequency of holiday trips during the past twelve months (p < 0.05) remained significantly associated with risk of overall overweight after adjustment for confounding variables [see Additional file 1]. Risk of overall overweight increased as the educational level decreased. Compared to women who had made one or more holiday trips during the previous twelve months, women who had not made any holiday trips had an increased risk of overall overweight. Compared to women who never smoked, current smokers also had an increased risk of being overweight or obese, independently of other variables. In multivariate analysis, similar associations were obtained for overall obesity (data not shown).

### Central obesity and SES

In multivariate logistic regression analyses, occupation (p < 0.05) and frequency of holiday trips during the previous twelve months (p < 0.01) were associated with central obesity in men [see Additional file 2]. Concerning subjects classified into the management/intermediate professional category, the fact of being self-employed or a farmer increased the risk of central obesity. Men who did not take any holiday trips during the twelve preceding months also had an increased risk of central obesity. In women, after adjustment for confounding factors, educational level (p < 10^-3^) and frequency of holiday trips during the past twelve months (p < 10^-3^) remained independently associated with central obesity [see Additional file 2]. Risk of central obesity increased when the educational level decreased, and women who did not take any holiday trip during the previous twelve-month period had an increased risk of central obesity compared to women who had made at least one holiday trip.

## Discussion

The ENNS survey provides comprehensive recent data on overweight and obesity in adult populations living in mainland France, based on measured anthropometric data. Overall, almost half (49.3%) of adults aged 18–74 were overweight or obese, and 16.9% were obese. The risk of overall and central overweight or obesity varied across SES categories both in men and women, but associations between overweight/obesity and SES were different between men and women. In men, overall and central overweight and obesity were inversely associated with occupational status, while central obesity was increased among men who had not taken a holiday trip during the past twelve months. In women, risk of overall and central overweight and obesity were higher among those with a lower education level and among women who had not taken any holiday trip during the past twelve months.

Previous estimations of prevalence of overweight and obesity available for French adults at the national level had been mainly based on self-reported data [3]. Underestimation of the prevalence of overweight and obesity due to underreporting of weight and overreporting of height is well documented in France [14], as in other industrialized countries [15-18]. Overweight and obesity estimated from ENNS were lower than those reported among adults in the USA (31.1% obesity in men and 32.2% in women) [19], Canada (22.9% in men and 23.2% in women) [20] and England (22.7% in men and 32.2% in women) [21]. They were close to those reported in Norway (15.5% obesity in men and 21.0% in women) [22], Spain (56.2% overweight or obesity in men and 40.9% in women) [23] and Italy (31.3% overweight in adults and 8.2% obesity) [24], although estimations for Spain and Italy based on self-reported data were underestimated. ENNS data confirm that France, like several neighbouring western European countries, is less strongly affected by overweight and obesity than the UK and eastern Europe countries [24].

The association between overweight/obesity and different SES dimensions (educational level, occupation, frequency of holiday trips), independently of confounding factors, suggests different underlying mechanisms. When possible, SES is generally measured by occupation, educational level and income [7,8], but due to limited availability of data, only a few surveys include all of these together. Occupation is considered to reflect job control, and a low employment position. Besides, a low job control has been shown to be associated with less leisure-time and physical activities [25-27]. A low job level may also be associated with higher exposure to work stress, leading to perturbed cortisol secretion and increased risk of overweight or obesity [28]. Educational level is considered to influence obesity-related health behaviour, such as specific dietary pattern, physical exercise, smoking habits, alcohol consumption and health- and nutrition-related knowledge and beliefs [4]. In British adults [29], weight control attitudes and practices were found to be more frequent in higher SES than in lower SES. Lower income may limit access to healthy foods (high-energy-density food is usually cheaper than healthy food) [30] and participation in leisure-time physical activities such as sports. Although, occupation, education level and income are not completely independent, it is of interest to analyze these three SES dimensions together [31].

Compared to occupation and income, which may fluctuate during a lifetime, education is assumed to be stable throughout life and to partly reflect childhood socioeconomic conditions. In the GLOBE longitudinal study [32], both childhood and adulthood socioeconomic deprivation increase the risk of overweight and obesity in adult women, whereas only adult SES influences the risk of overweight and obesity during adulthood in men. This observation is consistent with our data showing an association between education level and body mass observed among women, while there was no association between education and overweight/obesity among men in the final model.

Since publication of the seminal review of Sobal and Stunkard [5], several cross-sectional and longitudinal studies have shown a more consistent relationship between SES and overweight/obesity in women than in men. In particular, an inverse association has been observed between educational level and obesity in women in industrialized countries [4,8,26,33]. However, the relationship between SES and obesity remains complex and unclear [34,35], as SES influences overweight and obesity, while obesity influences SES, and still other factors may influence both obesity and SES. Disapproval of the obese may be frequent among women, and several surveys have shown that thinner women are more likely to experience personal, economic and educational success during their lifetime than their obese counterparts [26,36,37].

The absence of an association between occupation and overweight and obesity in women could be partly explained by differences in physical demands in men and women, with low-status jobs and manual occupations being considered as more sedentary for women than for men [8]. In contrast, among men, physical demands of low-status jobs could also protect against overweight/obesity, and could obscure the association between occupation and risk of becoming overweight or obese, in that the consequences of unhealthier lifestyle habits such as smoking, alcohol consumption and little leisure-time physical activity may thus be attenuated.

The risk of overweight and obesity was higher among subjects who did not take a holiday trip during the past twelve months (except for overall overweight and obesity in men). The association appears more clear-cut when using central obesity rather than overall overweight and obesity. BMI and WC measure different aspects of obesity and may have a different discriminatory function in terms of lifestyle factors [38]. BMI may not be the most relevant indicator of overweight and obesity, particularly in men. Due to physical demands related to low-status jobs, men in low-status jobs may, for a similar BMI level, have more muscle mass than men who occupy high-status positions [39]. In contrast, when adiposity is assessed through WC and central obesity, the association between overweight/obesity and low income, measured through the frequency of holiday trips, becomes significant and independent of other variables, both in men and women.

Although the rate of participation in the food consumption survey was about 60.0% and could be considered acceptable, participation in a health examination survey was lower (46.3%). The rather small sample size, particularly for men, may have limited the power of the study. Moreover, the initial sample was also characterized by underrepresentation of young adults and subjects with a lower education level, suggesting a participation bias [10]. Certain characteristics, such as interest in nutrition, healthy behaviour and health status, may have influenced participation in the survey. The participation bias was taken into account by a calibration procedure according to national census data on age, education level and the presence, or not, of at least one child in the household. Moreover, the proportion of subjects who did not take a holiday trip during the past twelve months and distribution according to birthplace, marital status and area of residence were mostly similar in the weighted sample and in the national census data, despite a few differences in age range. The potential distortion between the distribution according to occupation was not sufficiently important to substantially modify the estimated association between SES and outcomes. Nevertheless, the survey was based on national recruitment, and analyses were based on measured anthropometric data collected by trained physicians, nurses and dieticians according to WHO protocols. The validity of health examinations was greatly improved by carrying out home visits by nurses. Few social and demographic data were missing and risk of misclassification of SES variables was limited. The frequency of holiday trips during the twelve past months, strongly associated with family income [12], is easier to collect during a face-to-face interview, as the French population is highly unlikely to reveal its income. Use of occupational status of the head of the household, rather than the participant's own occupation, in multivariate logistic regression analyses led to the same conclusions for women (data not shown).

## Conclusion

In France, the prevalence of overweight and obesity is low compared to that in the USA, Canada, the UK and Eastern Europe countries. However, variations in the prevalence of overweight and obesity across SES are measurable both in men and women. In industrialized countries facing a rapid increase in overweight and obesity, such as the USA, the association between SES and obesity tends to decrease over time [40]. In the USA, socio-environmental phenomena such as increased serving sizes may be responsible for the increased prevalence of overweight and obesity, whereas individual characteristics, like SES, seem to be of less importance [40-42]. This is not the case in France, where lifestyle, healthier culinary habits and health attitudes, mainly depending on SES, still appear to play a role in the prevalence of obesity [2]. These results are important in defining new strategies for at-risk subpopulations, as defined by the PNNS.

## Competing interests

The authors declare that they have no competing interests.

## Authors' contributions

MV contributed to data collection management, conducted statistical analyses and wrote the manuscript. AM, AO, CD and ES contributed to data collection, monitoring and statistical analyses. BS and VD contributed to data collection management and statistical analyses, and revised the manuscript. SH contributed to protocol conception and revised the manuscript. KC conceived the protocol, managed data collection and revised the manuscript. All authors read and approved the final manuscript.

## Pre-publication history

The pre-publication history for this paper can be accessed here:



## Supplementary Material

Additional file 1 **Socioeconomic factors associated with overall overweight and obesity (BMI ≥ 25.0) in men and women, the French Nutrition and Health Survey (ENNS 2006–2007)**. The data provided represent logistic regression analyses carried out to investigate the association between SES marker and overall overweight and obesity. Click here for file

Additional file 2 **Socioeconomic factors associated with central overweight and obesity estimated through WC (♂: WC ≥ 94 cm, ♀: WC ≥ 80 cm), the French Nutrition and Health Survey (ENNS 2006–2007)**. The data provided represent logistic regression analyses carried out to investigate the association between SES marker and central overweight and obesity. Click here for file
