# Supplementary material for: Association of socioeconomic status with overall overweight and central obesity in men and women: the French Nutrition and Health Survey 2006
Source: BMC Public Health. 2009 Jul 2;9:215. doi: 10.1186/1471-2458-9-215 (PMC2714511; doi:10.1186/1471-2458-9-215)
Supplement: Additional file 1 — Socioeconomic factors associated with overall overweight and obesity (BMI ≥ 25.0) in men and women, the French Nutrition and Health Survey (ENNS 2006–2007). The data provided represent logistic regression analyses carried out to investigate the association between SES marker and overall overweight and obesity. [file 1471-2458-9-215-S1.doc]

**Table 2. Socioeconomic factors associated with overall overweight and obesity (BMI ≥ 25.0) in men and women, the French Nutrition and Health Survey (ENNS 2006-2007).**

|  | Men | | | | |  | Women | | | | |
| --- | --- | --- | --- | --- | --- | --- | --- | --- | --- | --- | --- |
|  | Univariate | |  | Multivariate | |  | Univariate | |  | Multivariate | |
|  | OR | CI_95_ |  | OR | CI_95_ |  | OR | CI_95_ |  | OR | CI_95_ |
| Age in years | **1.05** | **1.03-1.06** |  | **1.05** | **1.03-1.07** |  | **1.03** | **1.02-1.04** |  | **1.02** | **1.01-1.03** |
| Marital status |  |  |  |  |  |  |  |  |  |  |  |
| Married / living together | 1.00 | - |  |  |  |  | 1.00 | - |  |  |  |
| Single | **0.35** | **0.21-0.59** |  |  |  |  | **0.54** | **0.31-0.93** |  |  |  |
| Separated / divorced / widowed | 0.74 | 0.41-1.36 |  |  |  |  | **1.47** | **1.06-2.06** |  |  |  |
| Occupation |  |  |  |  |  |  |  |  |  |  |  |
| Management / intermediate profession | 1.00 | - |  | 1.00 | - |  | 1.00 | - |  |  |  |
| Self-employed / farmers | **2.21** | **1.00-4.89** |  | **2.15** | **1.07-4.32** |  | 2.44 | 0.98-6.04 |  |  |  |
| Manual workers / employees | 1.23 | 0.79-1.91 |  | 1.36 | 0.86-2.16 |  | **1.87** | **1.28-2.74** |  |  |  |
| Retired | **2.91** | **1.78-4.76** |  | 0.94 | 0.48-1.83 |  | **3.36** | **2.25-5.00** |  |  |  |
| Homemakers, disabled persons, others | 0.50 | 0.23-1.06 |  | 0.97 | 0.43-2.18 |  | **1.64** | **1.03-2.62** |  |  |  |
| Education level |  |  |  |  |  |  |  |  |  |  |  |
| University | 1.00 | - |  |  |  |  | 1.00 | - |  | 1.00 | - |
| High school | 1.47 | 0.87-2.46 |  |  |  |  | **1.56** | **1.01-2.42** |  | **1.55** | **1.01-2.39** |
| Secondary school | 1.19 | 0.80-1.77 |  |  |  |  | **2.78** | **1.93-3.99** |  | **2.46** | **1.68-3.61** |
| Primary school | **2.63** | **1.31-5.29** |  |  |  |  | **3.96** | **2.58-6.08** |  | **2.08** | **1.26-3.41** |
| Holiday trip during the past 12 months |  |  |  |  |  |  |  |  |  |  |  |
| Yes | 1.00 | - |  |  |  |  | 1.00 | - |  | 1.00 | - |
| No | 1.08 | 0.71-1.62 |  |  |  |  | **1.61** | **1.19-2.18** |  | **1.39** | **1.00-1.93** |
| Area of residence (%) |  |  |  |  |  |  |  |  |  |  |  |
| Rural | 1.00 | - |  |  |  |  | 1.00 | - |  | 1.00 | - |
| [2,000;20,000[ | 1.35 | 0.75-2.41 |  |  |  |  | 0.73 | 0.47-1.14 |  | 0.73 | 0.46-1.17 |
| [20,000;100,000[ | 0.92 | 0.48-1.78 |  |  |  |  | **0.59** | **0.38-0.92** |  | 0.63 | 0.39-1.01 |
| [100,000;2,000,000[ | **0.52** | **0.31-0.85** |  |  |  |  | **0.45** | **0.31-0.65** |  | **0.59** | **0.41-0.87** |
| Paris Area | 0.69 | 0.36-1.33 |  |  |  |  | 0.78 | 0.46-1.30 |  | 1.27 | 0.74-2.17 |
| Alcohol consumption (%) |  |  |  |  |  |  |  |  |  |  |  |
| Moderate | 1.00 | - |  |  |  |  | 1.00 | - |  |  |  |
| Abstainer | 0.70 | 0.40-1.25 |  |  |  |  | 0.90 | 0.65-1.25 |  |  |  |
| High | 1.38 | 0.83-2.28 |  |  |  |  | 0.98 | 0.55-1.76 |  |  |  |
| Smoking habits |  |  |  |  |  |  |  |  |  |  |  |
| Never-smoker | 1.00 | - |  |  |  |  | 1.00 | - |  | 1.00 | - |
| Current smoker | 0.64 | 0.40-1.03 |  |  |  |  | **0.47** | **0.32-0.67** |  | **0.57** | **0.39-0.84** |
| Former smoker | **1.95** | **1.21-3.17** |  |  |  |  | **0.70** | **0.49-0.99** |  | 0.74 | 0.52-1.07 |
