# Supplementary material for: Association of socioeconomic status with overall overweight and central obesity in men and women: the French Nutrition and Health Survey 2006
Source: BMC Public Health. 2009 Jul 2;9:215. doi: 10.1186/1471-2458-9-215 (PMC2714511; doi:10.1186/1471-2458-9-215)
Supplement: Additional file 2 — Socioeconomic factors associated with central overweight and obesity estimated through WC (♂: WC ≥ 94 cm, ♀: WC ≥ 80 cm), the French Nutrition and Health Survey (ENNS 2006–2007). The data provided represent logistic regression analyses carried out to investigate the association between SES marker and central overweight and obesity. [file 1471-2458-9-215-S2.doc]

**Table 3. Socioeconomic factors associated with central overweight and obesity estimated through WC (♂: WC ≥ 94 cm, ♀: WC ≥ 80 cm), the French Nutrition and Health Survey (ENNS 2006-2007).**

|  | Men | | | | |  | Women | | | | |
| --- | --- | --- | --- | --- | --- | --- | --- | --- | --- | --- | --- |
|  | Univariate | |  | Multivariate | |  | Univariate | |  | Multivariate | |
|  | OR | CI_95_ |  | OR | CI_95_ |  | OR | CI_95_ |  | OR | CI_95_ |
| Age in years | **1.06** | **1.05-1.08** |  | **1.08** | **1.05-1.10** |  | **1.04** | **1.03-1.06** |  | **1.03** | **1.02-1.05** |
| Marital status |  |  |  |  |  |  |  |  |  |  |  |
| Married / living together | 1.00 | - |  |  |  |  | 1.00 | - |  |  |  |
| Single | **0.26** | **0.14-0.47** |  |  |  |  | **0.43** | **0.26-0.73** |  |  |  |
| Separated / divorced / widowed | 1.25 | 0.68-2.27 |  |  |  |  | 1.17 | 0.83-1.64 |  |  |  |
| Occupational status |  |  |  |  |  |  |  |  |  |  |  |
| Management / intermediate profession | 1.00 | - |  | 1.00 | - |  | 1.00 | - |  |  |  |
| Self-employed / farmers | **2.58** | **1.20-5.54** |  | **2.45** | **1.19-5.07** |  | **2.83** | **1.13-7.14** |  |  |  |
| Manual workers / employees | 1.26 | 0.79-2.01 |  | 1.31 | 0.78-2.19 |  | **2.19** | **1.50-3.19** |  |  |  |
| Retired | **3.98** | **2.46-6.45** |  | 0.74 | 0.39-1.42 |  | **4.38** | **2.90-6.61** |  |  |  |
| Home makers, disabled persons, others | 0.75 | 0.34-1.66 |  | 1.63 | 0.68-3.93 |  | **1.80** | **1.13-2.86** |  |  |  |
| Education level |  |  |  |  |  |  |  |  |  |  |  |
| University | 1.00 | - |  |  |  |  | 1.00 | - |  | 1.00 | - |
| High school | 1.02 | 0.59-1.76 |  |  |  |  | 1.50 | 0.99-2.30 |  | 1.42 | 0.93-2.17 |
| Secondary school | 1.35 | 0.90-2.02 |  |  |  |  | **3.43** | **2.40-4.90** |  | **2.56** | **1.78-3.68** |
| Primary school | **3.62** | **1.88-6.97** |  |  |  |  | **5.68** | **3.59-8.98** |  | **2.57** | **1.54-4.29** |
| Holiday trip during the past 12 months |  |  |  |  |  |  |  |  |  |  |  |
| Yes | 1.00 | - |  | 1.00 | - |  | 1.00 | - |  | 1.00 | - |
| No | **1.86** | **1.23-2.80** |  | **1.93** | **1.24-2.99** |  | **2.19** | **1.59-3.01** |  | **1.90** | **1.34-2.71** |
| Area of residence (%) |  |  |  |  |  |  |  |  |  |  |  |
| Rural | 1.00 | - |  |  |  |  | 1.00 | - |  |  |  |
| [2,000-20,000[ | 1.33 | 0.76-2.32 |  |  |  |  | 0.83 | 0.52-1.32 |  |  |  |
| [20,000-100,000[ | 0.95 | 0.50-1.81 |  |  |  |  | 0.68 | 0.43-1.08 |  |  |  |
| [100,000-2,000,000[ | **0.49** | **0.30-0.81** |  |  |  |  | **0.54** | **0.37-0.79** |  |  |  |
| Paris | 0.57 | 0.30-1.06 |  |  |  |  | 0.68 | 0.40-1.14 |  |  |  |
| Alcohol consumption (%) |  |  |  |  |  |  |  |  |  |  |  |
| Moderate | 1.00 | - |  |  |  |  | 1.00 | - |  |  |  |
| Abstainer | 0.83 | 0.46-1.50 |  |  |  |  | 0.98 | 0.70-1.37 |  |  |  |
| High | 1.57 | 0.97-2.53 |  |  |  |  | 0.657 | 0.64-2.03 |  |  |  |
| Smoking habits |  |  |  |  |  |  |  |  |  |  |  |
| Never-smoker | 1.00 | - |  |  |  |  | 1.00 | - |  |  |  |
| Current smoker | 0.72 | 0.44-1.19 |  |  |  |  | **0.68** | **0.48-0.97** |  |  |  |
| Former smoker | **2.06** | **1.29-3.29** |  |  |  |  | 0.78 | 0.55-1.11 |  |  |  |
